# A “Skylight” Simulator for HWIL Simulation of Hyperspectral Remote Sensing

**DOI:** 10.3390/s17122829

**Published:** 2017-12-06

**Authors:** Huijie Zhao, Bolun Cui, Guorui Jia, Xudong Li, Chao Zhang, Xinyang Zhang

**Affiliations:** School of Instrumentation Science & Opto-electronics Engineering, Key Laboratory of Precision Opto-Mechatronics Technology, Beihang University, Ministry of Education, 37# Xueyuan Road, Haidian District, Beijing 100191, China; hjzhao@buaa.edu.cn (H.Z.); boluncui@buaa.edu.cn (B.C.); xdli@buaa.edu.cn (X.L.); xyliang@buaa.edu.cn (C.Z.); xy_zhang@buaa.edu.cn (X.Z.)

**Keywords:** HWIL, simulation, skylight, mineral mapping, sensor test, spectral measurement, remote sensing experiment

## Abstract

Even though digital simulation technology has been widely used in the last two decades, hardware-in-the-loop (HWIL) simulation is still an indispensable method for spectral uncertainty research of ground targets. However, previous facilities mainly focus on the simulation of panchromatic imaging. Therefore, neither the spectral nor the spatial performance is enough for hyperspectral simulation. To improve the accuracy of illumination simulation, a new dome-like skylight simulator is designed and developed to fit the spatial distribution and spectral characteristics of a real skylight for the wavelength from 350 nm to 2500 nm. The simulator’s performance was tested using a spectroradiometer with different accessories. The spatial uniformity is greater than 0.91. The spectral mismatch decreases to 1/243 of the spectral mismatch of the Imagery Simulation Facility (ISF). The spatial distribution of radiance can be adjusted, and the accuracy of the adjustment is greater than 0.895. The ability of the skylight simulator is also demonstrated by comparing radiometric quantities measured in the skylight simulator with those in a real skylight in Beijing.

## 1. Introduction

Simulation is an indispensable process in the calibration and validation of instruments or analysis of algorithms. It is also an important way to analyze the characteristics of a target in specific circumstances. Over the last two decades, digital simulation has become the major approach to simulating hyperspectral imaging along the sun–target–observer image chain [1,2,3,4]. In the process of digital simulation, solar irradiance, skylight, and reflected background radiance are considered to illuminate the target. Then, the radiance to the sensor is calculated as a combination of directly reflected radiance and upwelled radiance of atmosphere [5,6,7]. Lastly, the sensor characteristics are described using common models. This is a good approach to simulating images in all kinds of radiation and imaging geometric conditions. However, its performance is limited by the knowledge of characteristics of the target, environment, and sensor.

Although the precision reflectance model of buildings and canopies [8,9] and the mixing model of minerals (such as the Hapke model [10,11]) have been developed, and the precision model of a spectrometer can also be specifically built [12], it is still important to measure reflected radiance in a hardware-in-the-loop (HWIL) simulation when researching the influence of environment, such as heavy metal toxicity in plants, nonlinear mixing of minerals, or weathered minerals [13,14,15,16,17,18,19,20,21,22]. The traditional approaches include conducting flight campaigns over experimental sites [13,14,15] and experiments in labs [16,17,18,19,20,21,22]. The flight campaign approach has several shortcomings such as the great expense, time consumption, the complexity and difficulty of making simultaneous in-situ measurements of atmospheric parameters and ground reflective characteristics. The result of using a flight campaign is also limited by many environmental conditions such as solar zenith, weather, and visibility.

Therefore, most experiments for researching or instrument testing are accomplished in laboratories by measuring the spectral reflectance of the target. The common light source illuminating targets are halogen lamps [16,17,18,19] or halogen lamps combined with a collimator or integrating sphere [20,21,22]. In this context, the indoor experiment faces some challenges. Firstly, the irradiance illuminated on the target can hardly simulate the geometric characteristics of solar irradiance and skylights. Secondly, the spectral characteristic of halogen lamps is quite different from that of solar irradiance or skylights [23].

In order to simulate the remote multispectral imaging process indoors, an HWIL simulation facility named Imagery Simulation Facility (ISF) was developed by ITEK Optical System (Lexington, MA, USA) [24], which includes both solar and skylight simulators. However, the spectral characteristic of the tungsten lamps selected for the skylight simulator was quite different from real skylights. Therefore, they are respectively filtered with several different filters for the spectral range from 400 nm to 2500 nm, or replaced by a different kind of lamp with accurate spectral characteristics from 400 nm to 800 nm for color film imaging simulation [24]; this results in either of the following two problems. First, the spectral isotropy over the hemisphere is decreased by different filters. Second, the facility can only work in the visible region. Except for the above problems, the change in skylight irradiance with different solar zeniths was not reported. Therefore, the ISF facility cannot be used to test a hyperspectral system or algorithm, especially for studies on mineral identification, which are mostly based on spectral features in the short-wave infrared (SWIR) region.

In this paper, a hemispherical lamp array with 80 identical lamps is designed as a skylight simulator for the indoor simulation of hyperspectral remote sensing. A metal-halide lamp is chosen as the light source in the simulator and designed to point to the center of the array. The spatial distribution of lamps is modeled and tested considering the slightly different performances among individual lamps. A series of experiments are conducted to test the performance of the simulator. As the result, the spatial uniformity of irradiance is greater than 0.91. The spectral mismatch is about 1/243 that of the ISF. The spatial distribution of radiance can be adjusted, and the accuracy of the adjustment is greater than 0.895.

Section 2 analyzes the characteristics of the skylight and draws the requisite performance of the skylight simulator. Section 3 indicates the development of the skylight simulator. In Section 4, the accuracy of the simulator is tested. Conclusions are drawn in Section 5.

## 2. Theory

**Figure 1:**
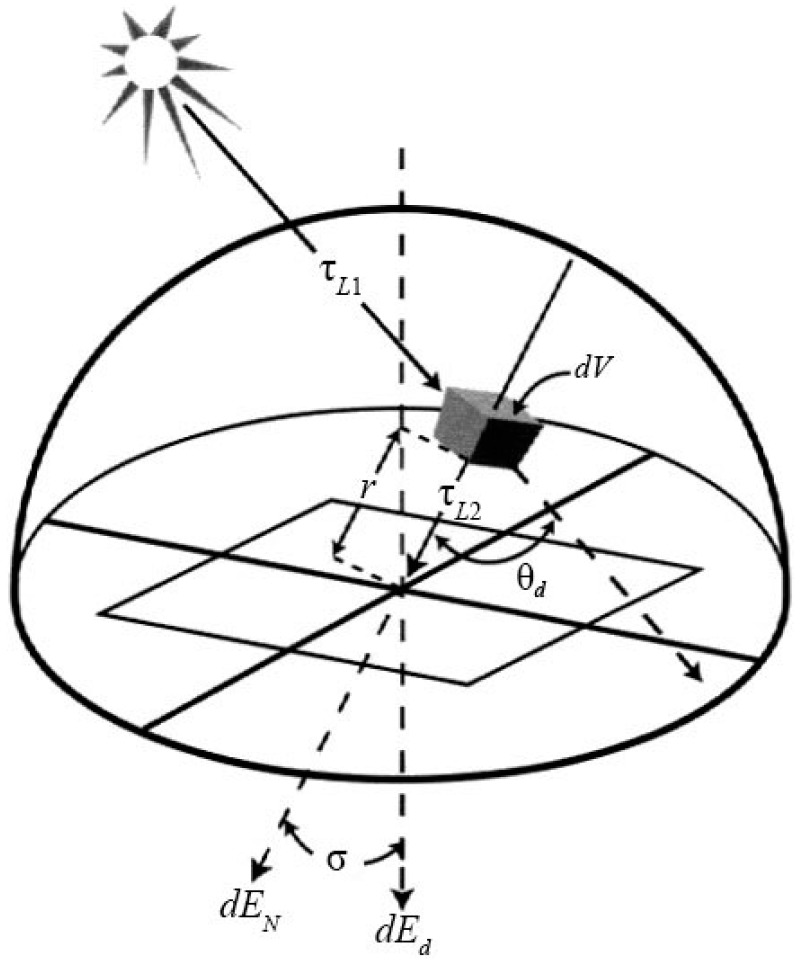
Contribution to the downwelled irradiance from a unit volume.

The spectral irradiance onto the surface, $E_{\tau \lambda }$, can be divided into three parts including direct solar irradiance $E_{s\lambda }^{\prime }$, skylight irradiance $E_{d\lambda }$, and background radiance $L_{b\lambda avg}$ [25]:

(1) $$E_{\tau \lambda }=E_{s\lambda }^{\prime }\cos\sigma^{\prime }\tau_{1}(\lambda )+FE_{d\lambda }+(1-F)L_{b\lambda avg}\pi$$

where F is the fraction of the hemispherical sky that could be seen from the target, $\sigma^{\prime }$ is the zenith of the target, and $\tau_{1}(\lambda )$ is the transmission from solar to the scattering volume. As shown in Figure 1 [25], the downwelled irradiance (skylight) $E_{d\lambda }$ could be modeled as the integration of the directional radiance from the scattering of solar light by a small unit volume over the hemisphere area above the target [25]:

(2) $$E_{d\lambda }=\int \frac{E_{s\lambda }^{\prime }\tau_{L1}(\lambda )\tau_{L2}(\lambda )\beta_{sca}(\lambda ,\theta_{d})\cos\sigma dV}{r^{2}}$$

where σ is the angle between the normal to the target and the ray from the volume dV, *r* is the distance from the target to the volume which will be integrated from the target to the top of atmosphere (TOA), and $\tau_{L1}(\lambda )$ and $\tau_{L2}(\lambda )$ are the transmissions along the paths L1 and L2. This reveals that $E_{s\lambda }^{\prime }$ is attenuated through the sun–volume path L1, scattered in a deflection of $\theta_{d}$ onto the target, in the fraction of the angular scattering coefficient $\beta_{sca}(\theta_{d},\lambda )$, and attenuated again through the volume–target path L2.

Accordingly, the skylight generally keeps three characteristics. (a) The target is illuminated by a hemispherical skylight and the spatial radiation distribution is influenced by the solar direction. (b) The total irradiance varies with the atmospheric status. (c) The total spectral irradiance arriving at the ground is spatially uniform.

In order to achieve a group of standard spectra of irradiance, MODTRAN 4.1 (Spectral Sciences Inc., United States Air Force, USA) was used to calculate the radiance from different directions and irradiance onto the ground surface for different dates and times [26]. Ground locations were set at Hami, Xinjiang, China, which is a typical zone with many porphyry copper–gold mineralization subzones [27]. The parameters used in MODTRAN calculation are listed in Table 1.

**Figure 2:**
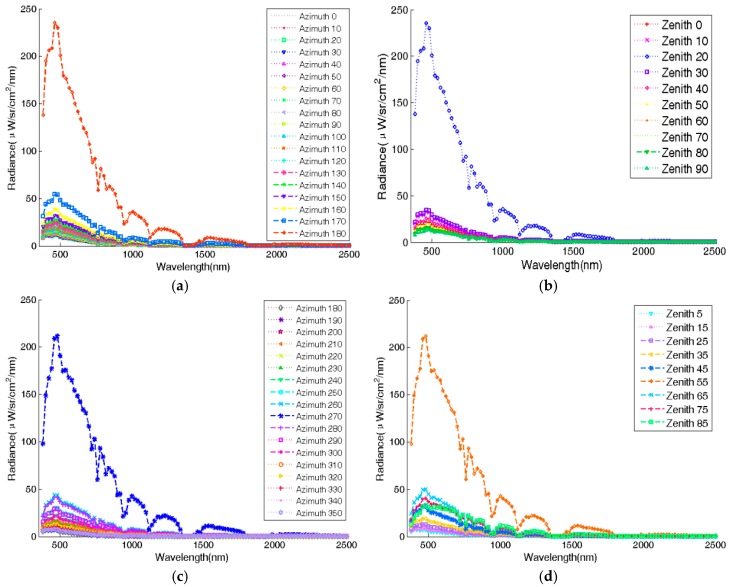
The radiance of the skylight: (**a**) The spectral radiance from zenith 20° and different azimuths at 12:00; (**b**) The spectral radiance from azimuth 170° and different zeniths at 12:00; (**c**) The spectral radiance from zenith 20° and different azimuths at 16:00; (**d**) The spectral radiance from azimuth 170° and different zeniths at 16:00.

As shown in Figure 2, the skylight irradiates the target from every direction and varies with incident angle, holding the same spectral characteristic. The radiance comes to the maximum in the solar direction (zenith 17°, azimuth 180°) and the minimum appears in the opposite direction of solar incidence, as shown in Figure 2a,b. The spectral isotropy of radiance is tested by calculating the spectral correlation coefficients between azimuthal-average spectral radiance with different zenith angles and between zenithal-average spectral radiance with different azimuth angles, respectively, as shown in Table 2 and Table 3. The Pearson correlation coefficient is calculated according to Equation (3), where $X_{m}\left(i\right)$ and $X_{n}\left(i\right)$ represent the radiances in band i with different zenith/azimuth angles respectively. As the correlation in Table 2 and Table 3 is greater than 0.980, the spectral characteristic of radiance can be summarized as stable with the change of zenith and azimuth. The radiance at 16:00 shows the same characteristic in Figure 2c,d (the solar zenith is 55° and azimuth is 180°). Caused by the change in solar direction, the spatial distribution of the radiance changed. The correlation of the max radiance, min radiance, and middle one at 12:00 and 16:00 is also calculated, as shown in Table 4. The spectral characteristic at different times can be defined as stable, because the correlation is greater than 0.957.

(3) $$Corr=\frac{E\left(\left(X_{m}\left(i\right)-E\left(X_{m}\left(i\right)\right)\right)\left(X_{n}\left(i\right)-E\left(X_{n}\left(i\right)\right)\right)\right)}{\sqrt{D\left(X_{m}\left(i\right)\right)}\sqrt{D\left(X_{n}\left(i\right)\right)}}$$

The stability of the spectral characteristic ensures the probability of adjusting the irradiance of the skylight simulator. It is obvious that the spatial uniformity of irradiance and spatial distribution of radiance onto the ground are two very important characteristics of the skylight. When adjusting the zenith distribution of the radiance, the difference in radiance between each target could be negligible. However, when adjusting the azimuth distribution of radiance, lamps need to point to different targets, respectively, to maintain the uniformity of irradiance, which will cause great differences in radiance on targets. As a result, the simulation of azimuth distribution of radiance is almost impossible. Therefore, the skylight simulator is supposed to be adjusted to simulate the zenith distribution of radiance, and maintain the spatial uniformity of spectral irradiance at the same time. The irradiance and the zenith distribution of the radiance would be similar to the MODTRAN-simulated result shown in Figure 2.

## 3. Instrument Description

### 3.1. Instrument Design

#### 3.1.1. Chosen Light Source

As mentioned above, adjusting the lamps with different filters will decrease the spectral isotropy of radiance. In addition, it is obvious that combining different kinds of bulbs into one set of reflector and fore-optics to act as a lamp in the lamp array is nearly impossible, even when not considering the increase in power cost. Therefore, the best way to simulate the spectral characteristics of a skylight would be to find a light source whose spectral irradiance on the target is most similar to a skylight and then calibrate it. In order to choose the best light source for the skylight simulator, the characteristics of four kinds of lamp were compared, i.e., LED lamp, tungsten halogen lamp, xenon lamp, and metal-halide lamp.

**Figure 3:**
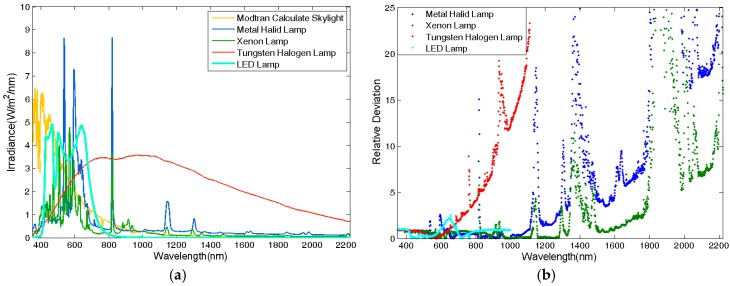
The radiances of different lamps compared with skylight radiance. (**a**) The spectra of lamps and (**b**) the relative deviations of lamps.

The spectral irradiance of these lamps, as shown in Figure 3a, was collected in a darkroom by using an ASD FieldSpec pro FR spectroradiometer (Analytical Spectral Devices, Inc., Longmont, Colorado) with a full-sky irradiance Remote Cosine Receptor (RCR) accessory. Their relative deviations from the skylight spectrum in band i are calculated with Equation (4) and plotted in Figure 3b. The spectral correlation between lamps and skylight is calculated with Equation (3) and listed in Table 5. It is obvious that the spectral irradiance of the LED lamp only distributes in the visible region. The relative deviation of the tungsten halogen lamp, which is equipped in ISF, is much greater than those of the metal-halide lamp and xenon lamp. A xenon lamp is a kind of high intensity discharge (HID) lamp, which is used as a cinema projector lamp, vehicle lamp, and solar simulator for photovoltaic cell testing [28,29,30,31,32]. However, there is no compact xenon light source with a wide beam. Most of the xenon bulbs available now are bare bulbs without a reflector or fore-optics. In order to improve the illuminating efficiency, the xenon bulbs must be combined with specific fore-optics and reflectors. However, the accuracy of assembly and the isotropy of the shape of free-form surface reflectors and Fresnel lenses are limited. Therefore, the compact metal-halide lamp is chosen to be the light source, so that we could select lamps performing uniformly enough from among industrial products.

By comparing the luminous efficacy, mean lumens, and energy consumption of several types of lamp produced by PHILIPS (Amsterdam, The Netherlands), OSRAM (Munich, Germany), and GE (East Cleveland, OH, USA), the PHILIPS CDM-RM Mini 20W/830 GX10 MR16 40D [33] was chosen at last.

(4) $$E\left(i\right)=\frac{X_{m}\left(i\right)-X_{0}\left(i\right)}{X_{0}\left(i\right)}$$

#### 3.1.2. Optical Model

**Figure 4:**
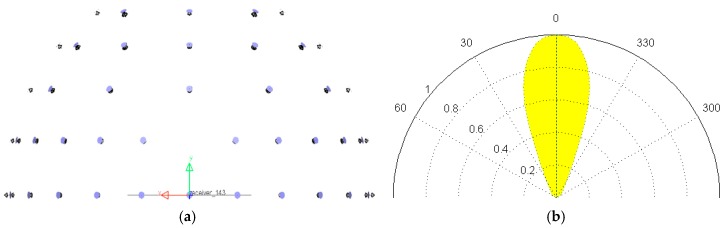
The model built by LightTools. (**a**) The spatial distribution of lamps in the hemispherical lamp array, and (**b**) the normalized intensity distributed by the zenith of one lamp.

The skylight simulator needs to simulate spatially uniform spectral irradiance, and can be adjusted to simulate different zenith distributions of radiance, as mentioned in Section 2. In order to simulate the spatial uniformity of spectral irradiance, the lamps are designed to be distributed as a circle and orient to the center of the hemisphere. The skylight illuminates the target from different directions over the whole hemisphere, so the skylight simulator is designed as a hemispherical lamp array. The lamps at the polar region of the hemisphere are omitted to leave more space for the spectrometer when performing the imaging simulation. In order to fit the irradiance on the illumination region with the result calculated by MODTRAN, a complicated model was developed with LightTools (Synopsys, Mountain View, CA, USA) [34], as shown in Figure 4. Table 6 lists parts of the parameters of the model. The optical characteristics of lamps, including the size, the shape of the beam, and the spectral radiant exitance, were carefully tested with a Luminance Meter and a Spectrometer. The radius of the skylight simulator is designed to be less than 3.3 m, considering the space of the lab.

As the simulator is supposed to adjust the zenith distribution of radiance, the distribution of the lamps, along with the zenith angle, needs to be carefully designed. As the reflectance or the bidirectional reflectance distribution function (BRDF) are described with irradiance—not radiance—on the target [25], it is better to define the performance of the skylight simulator with irradiance. In addition, it is obvious that the irradiance on the ground is much more available than radiance in a specific zenith region. The irradiance contributed by the skylight in specific zenith regions (every 15°) at different times is the integral of the radiance calculated using MODTRAN, as shown in Table 7.

The irradiance on the center of the ground illuminated by the lamps on a specific zenith can be described by Equation (5), where *n* is the number of lamps on a specific zenith, $I_{i}$ is the intensity of the lamp at solid angle $\Omega_{i}$, $\theta_{i}$ is the zenith angle, and A is the area on the ground. The irradiance contributed by the skylight from the zenith region $\left[\theta_{i}-0.5\Delta \theta ∼\theta_{i}+0.5\Delta \theta \right]$ is close to the irradiance illuminated by the lamp array on zenith $\theta_{i}$. The number of lamps *n* can be estimated with Equation (5), and the data are listed in Table 7.

(5) $$E_{i}=\frac{n\int I_{i}d\Omega_{i}}{\cos\theta_{i}dA}$$

At last, the number and distribution of lamps were designed as shown in Table 8 with three typical modes. Mode 1 is used when the solar zenith is less than 40°, Mode 2 is used when the solar zenith varies from 40° to 65°, and Mode 3 is designed to simulate skylight when the solar zenith is greater than 65°. Due to the regular interval of lamps, the skylight simulator can be adjusted to fit many more situations such as an atmosphere with a different extinction coefficient or atmosphere on different dates.

### 3.2. System Development

**Figure 5:**
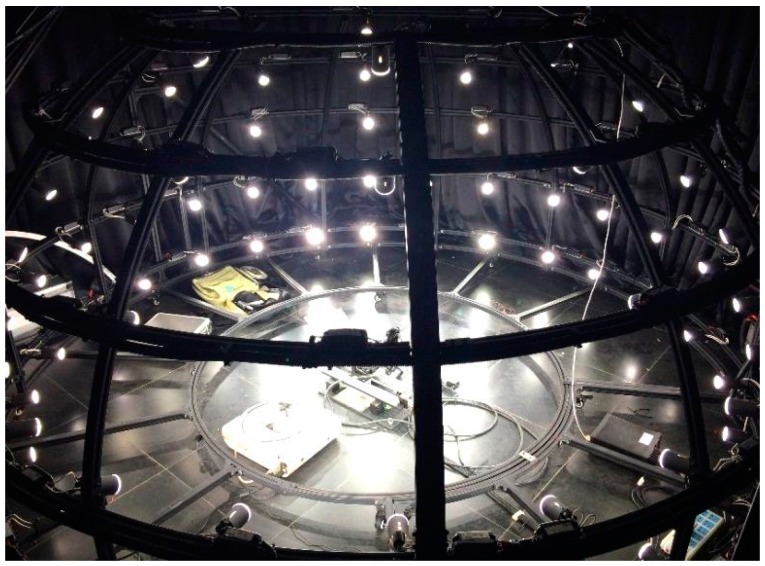
The working status of the skylight simulator.

In order to prevent the interference of illumination, the lab was built as a darkroom. All of the devices in the lab were painted matte black, which reduces the illumination reflected from the environment as much as possible. The brace of the lamp array was manufactured with aluminum profiles to give high rigidity. The curvature accuracy of the round components was carefully adjusted to be greater than 0.999. Each lamp with specific electronic ballast was measured as mentioned above and fixed according to the model designed by LightTools, as shown in Figure 5. All of the lamps were divided into several groups with RS-485 bus controllers connected separately. Each lamp could be controlled with an outside computer.

## 4. Performance Validation

In order to test the performance of the skylight simulator, a series of experiments were performed. The essential performance indicators, including the spatial uniformity, the spectral mismatch, and the accuracy of adjustment, were tested indoors. The experimental performance including the agreement of irradiance, the spatial distribution of radiance, the spectral characteristic, and the collected reflectance were tested by comparing the radiometric quantities collected in the field with those collected in the simulator.

### 4.1. Essential Performance

In order to test the spatial uniformity of the skylight simulator in each band, the illuminated plane (50 cm × 50 cm at the center of simulator) was divided into a 10 × 10 grid. An ASD Fieldspec Pro spectrometer equipped with an RCR accessory was used to collect the irradiance E(λ) on each node. The spatial uniformity was then calculated with Equation (6) according to the IEC 60904-9 standard [35]:

(6) $$U\left(\lambda \right)=1-\frac{E_{\max}\left(\lambda \right)-E_{\min}\left(\lambda \right)}{E_{\max}\left(\lambda \right)+E_{\min}\left(\lambda \right)}.$$

**Figure 6:**
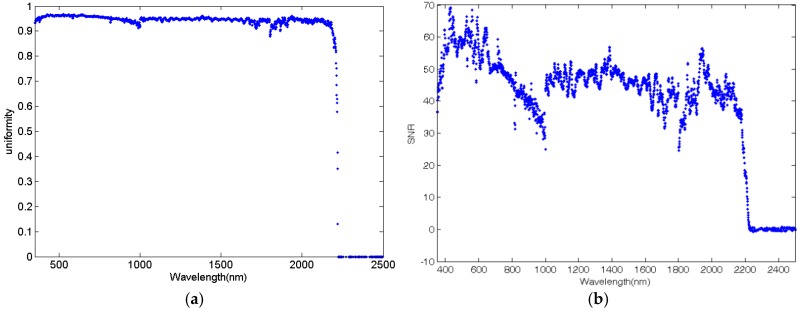
The spectral non-uniformity of the skylight simulator: (**a**) The spectral non-uniformity of the skylight simulator in 350–2500 nm spectral region and (**b**) the signal-to-noise ratio (SNR) of spectral irradiance collected with an ASD field spectrometer.

The results are shown in Figure 6a. The measured irradiance—and, hence, the uniformity—from 2200 nm to 2500 nm are invalid, because the transmittance of the RCR decreases substantially in this spectral range, which is revealed by the signal-to-noise ratio (SNR) of the collected irradiance calculated with Equation (7) and shown in Figure 6b. Generally, the uniformity is greater than 0.88 from 350 nm to 2200 nm. Moreover, the uniformity is greater than 0.91 in most bands. In a word, the performance of the skylight simulator is good enough for simulating the spatial uniformity of spectral irradiance, as mentioned in Section 2.

(7) SNR(λ)=∑nE(λ)n∑n(E(λ)−∑nE(λ)n)2n

**Figure 7:**
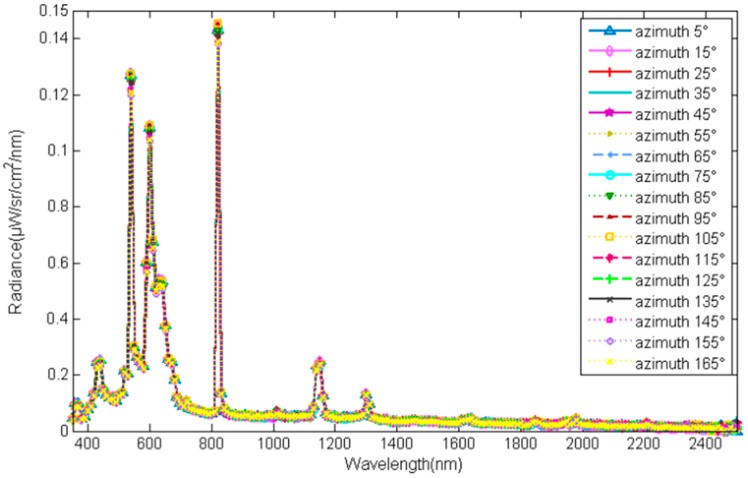
The spectral radiance reflected by the standard target.

The spectral isotropy over the hemisphere of the skylight simulator was tested by collecting the radiance reflected by a target with specific zenith angle facing different directions. The zenith of the target was 30°, and the azimuth of it was set to change from 0° to 180° in intervals of 5°. The radiance is shown in Figure 7 (part of data), and the correlation between them was calculated to be greater than 0.9995. Because only one kind of lamp was used, the spectral mismatch of the skylight simulator is similar to the performance of a single lamp shown in Figure 3, even if the spectral characteristic of each lamp is not exactly the same.

This high spectral isotropy performance indicates that the facility can be adjusted without decreasing spectral isotropy, which is defined as the first characteristic of a skylight in Section 2.

**Figure 8:**
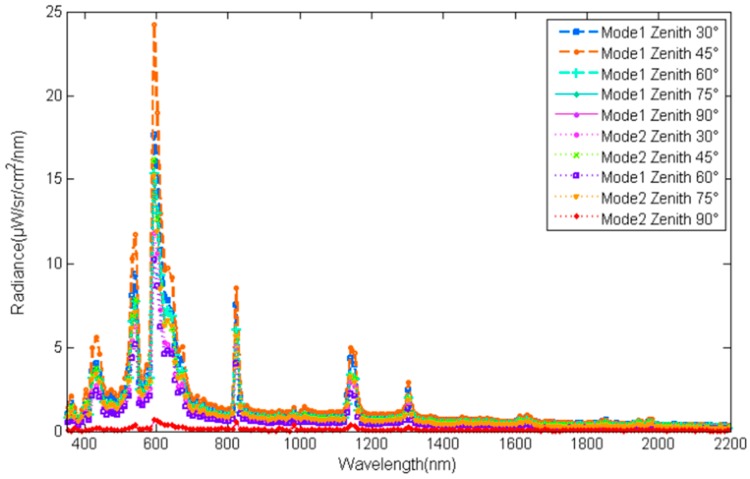
The spectral irradiance contributed by different zenith regions.

According to Equation (5), the irradiance contributed by different zenith regions can be calculated using the irradiance of lamps at different zeniths. Therefore, the accuracy of adjusting the irradiance contribution to the simulated skylight at three different times of the day, i.e., corresponding to three different solar zenith angles, can be measured with a spectrometer, as shown in Figure 8. Comparing the irradiance in Table 9, the relative deviation of adjusting was calculated to be less than 0.105 with Equation (4), where $X_{m}$ is the simulated irradiance and $X_{0}$ is the irradiance calculated with MODTRAN. The spatial distribution accuracy of radiance was evaluated with the RMSRE (root mean square relative error) of irradiance in different zeniths. The RMSRE is calculated using Equation (8), where $X_{i}$ is the simulated irradiance in zenith i and $X_{i}^{\prime }$ is the irradiance calculated with MODTRAN. The results are about 0.189 in Mode 1, 0.259 in Mode 2, and 0.198 in Mode 3. Therefore, the irradiance on the ground and the spatial radiance can be adjusted with accuracy greater than 0.895 and 0.741, respectively. The first and second characteristics described in Section 2 can therefore be simulated by the facility.

(8) $$RMSRE=\sqrt{\frac{\sum_{i=1}^{n}{\left(\frac{X_{i}-X_{i}^{\prime }}{X_{i}^{\prime }}\right)}^{2}}{n}}$$

### 4.2. Experimental Performance

**Figure 9:**
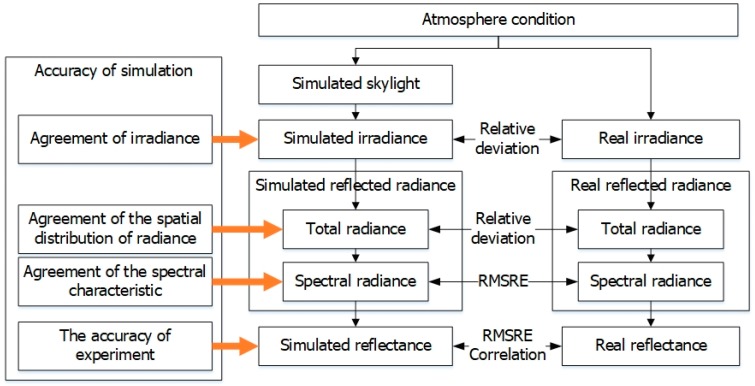
The flow chart of the experiment. RMSRE: root mean square relative error.

In order to validate the ability of the skylight simulator, an experiment was performed on 24 August 2017 in Beijing, as shown in Figure 9. The skylight simulator was adjusted to simulate the irradiance when the solar zenith angle is 28.9°. The total irradiances collected both in the field and in the skylight simulator were compared to test the agreement at the irradiance level. The total radiance reflected by the target both in the field and the simulator were also compared using the metric RMSRE to test the agreement at the spectral characteristic level. The accuracy of the experiment was tested by comparing the reflectance of the target collected in the field and in the simulator.

**Figure 10:**
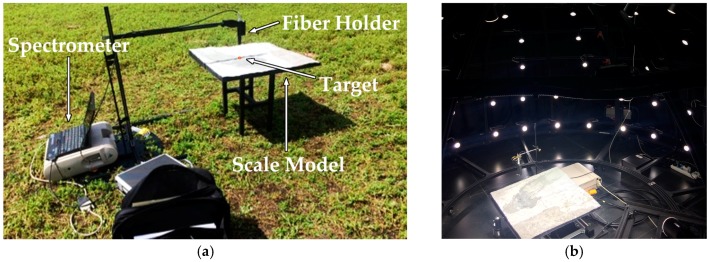
Photographs of the experiment: (**a**) collecting the reflected radiance of the target in the field and (**b**) collecting the reflected radiance in the skylight simulator.

Beijing is dominated by a warm, temperate, continental monsoon climate [36]. The MODTRAN atmosphere model is defined as mid-latitude summer [26]. The cloud cover is less than 15%, with the solar zenith at 28.9°. A scale model of the Cuprite mineral area in China and a geographical model including several slopes with different slope angles are placed on the ground with skylight illumination. The direct solar radiance is blocked by a black plastic fiber holder, as shown in Figure 10.

**Figure 11:**
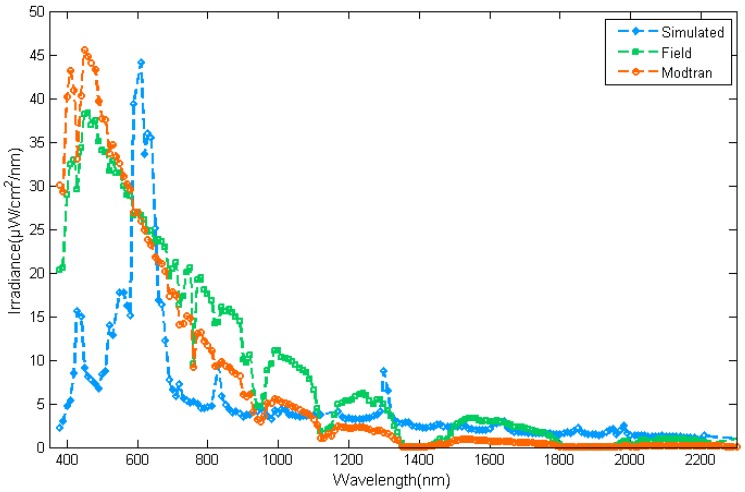
The irradiance of field-collected and simulated skylight (four peaks in the irradiance of the simulator have been removed as bad bands).

The irradiance of the real skylight was collected using an ASD Fieldspec Pro spectrometer with an RCR accessory as shown in Figure 11, with the total irradiance measured as 14,570 μW/cm^2^. Since the radiance of the simulator at zenith 0–30° is removed, the irradiance collected in the field was scaled to 11,214 μW/cm^2^ according to the irradiance simulated by MODTRAN. The relative deviation of the irradiance from the skylight simulator compared with the scaled field-collected irradiance is less than 0.021. The irradiance from the simulator in 350–600 nm is less than that collected in the field as a result of the limitation of the light source, as shown in Figure 11.

**Figure 12:**
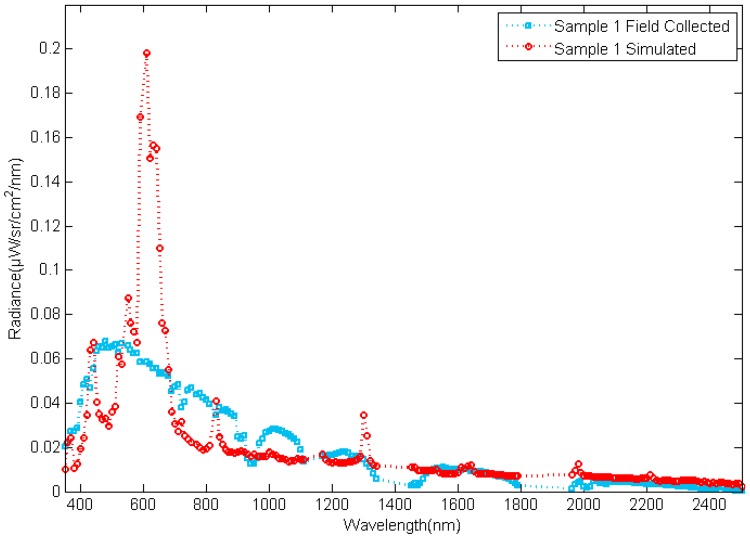
The radiance reflected by targets.

**Figure 13:**
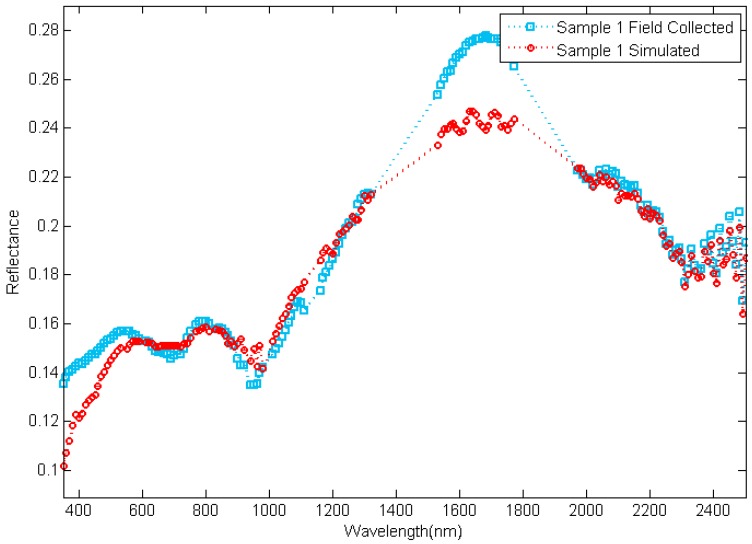
The reflectance collected in the field and in the skylight simulator.

A scaled model with different targets was used in the experiments. The targets on the model were made from several kinds of rock-forming minerals such as feldspar, pyroxene, chlorite, epidote, muscovite, olivine, crystal, kaolinite, calcite, serpentine, and so on. The radiance reflected by the targets and the reflectance of them were collected using a spectrometer with an FR 8 DEG Field-of-View Lens Fore optic [23] as shown in Figure 12 and Figure 13, respectively.

**Figure 14:**
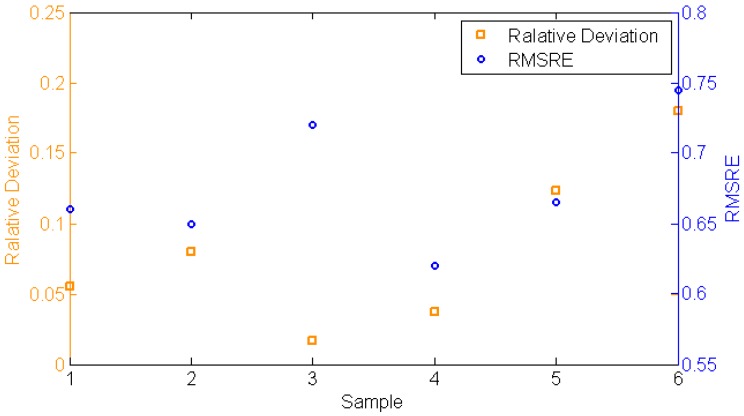
The comparison of radiance.

The radiance reflected by targets is related to the spatial distribution of the skylight radiance. The relative deviation of the total radiance was calculated with Equation (4) to be less than 0.184, as shown in Figure 14, where $X_{m}$ is the radiance collected in the simulator and $X_{0}$ is the radiance collected in the field. The RMSRE of the spectral radiance was calculated using Equation (8), where $X_{i}$ is the radiance in band i collected in the simulator and $X_{i}^{\prime }$ is the radiance in band i collected in the field. The RMSRE varies from 0.613 to 0.744, as shown in Figure 14. Accounting for the spectral mismatch of radiance in the 460–510 nm and 600–660 nm regions, the RMSRE is still high. However, the spectral mismatch can be calibrated with a spectral coefficient later for application to hyperspectral remote sensing as the deviation of most of the bands is low. The RMSRE of radiance simulated by halogen tungsten lamps is also calculated to be 170.7, which is much higher compared with that of the skylight simulator. The accuracy of the radiance simulation is improved significantly by choosing the lamps to be spectral matched.

**Figure 15:**
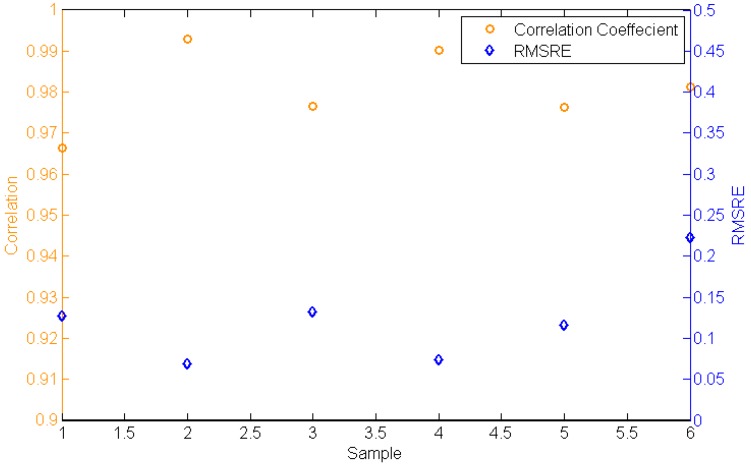
The comparison of reflectance.

As the information of the target is expressed as reflectance, the spectra of reflectance collected in different situations are compared in Figure 13. The RMSRE and the correlation of reflectance are shown in Figure 15. The RMSRE of reflectance is calculated with Equation (8), where $X_{i}$ is the reflectance in band i collected in the simulator and $X_{i}^{\prime }$ is the reflectance in band i collected in the field, and is less than 0.233. The correlation of reflectance is calculated with Equation (3), where $X_{m}(i)$ is the reflectance in band i collected in the simulator and $X_{n}(i)$ is the reflectance in band i collected in the field, and is greater than 0.966.

## 5. Conclusions

The skylight simulator is an important facility in the HWIL simulation of remote sensing, especially for hyperspectral remote sensing. The previous facilities, including the ISF developed by ITEK Optical System, are not applicable in a hyperspectral remote sensing experiment. The two main reasons for this are the mismatch of spectral characteristics and lack of ability of adjustment for the simulation of different scenes, because of the limitations of the light source and structure. A new facility was designed and developed with a new wide-beam metal-halide lamp and hemisphere structure to spatially and spectrally simulate skylight illumination.

The performance of the proposed skylight simulator was tested using a spectrometer with different accessories in different situations. The spatial uniformity of spectral irradiance is greater than 0.91 in almost all bands from 350–2200 nm. The spectral match of the simulator with the real skylight improves by about 243 times in the visible and near infrared (VNIR) and SWIR regions compared with a halogen-lamp-based system such as ISF. The spectral isotropy over the hemisphere is greater than 0.9995. The accuracy of adjusting the irradiance of the simulator is greater than 0.895 for three modes. The accuracy of irradiance distribution along different zeniths is greater than 0.741. 

To validate the spectral performance of the simulation, the spectra of irradiance, radiance reflected by targets, and reflectance of targets were collected with the skylight simulator and compared with those collected in the field. The relative deviation of irradiance is less than 0.021. The RMSRE of spectral radiance is 0.6–0.7 with the relative deviation of radiance less than 0.184. The RMSRE of spectral reflectance is less than 0.233, and the correlation is greater than 0.966. The spectral isotropy over the hemisphere is greater than 0.997.

All the above spectral accuracies in the 350–2500 nm region are about 243 times those of the latest facility. Simultaneously, the isotropy of the spectral characteristic is ensured, and the adjusting accuracies of irradiance and radiance are greater than 0.895 and 0.741, respectively.

Future work will be focused on developing new light sources working in 350–2500 nm to improve the accuracy of the spectral simulation, and, in particular, to decrease the mismatch of the spectral characteristic in the 350–600 nm region.

## Figures and Tables

**Table 1:** Parameters Used in MODTRAN Simulation.

Attribute	Value
Day of Year	1–365 (interval 15)
Local Time	10:00–14:00 (interval 1 h)
Viewing Zenith Angle (°)	0–90 (interval 5)
Viewing Azimuth Angle (°)	0–360 (interval 5)
Location	40° N, 94° E
Spectral Range (nm)	400–2500
Spectral Response Function	Gaussian of FWHM 10 nm
Spectral Sampling Interval (nm)	10
Model of atmosphere	Sub-Arctic Summer Mid-Latitude Summer
Model of Aerosol	Rural
Visibility	40 km

**Table 2:** The correlation between azimuthal-average spectral radiances and different zenith angles.

Zenith	0°	15°	30°	45°	60°	75°	90°
0°	1.000						
15°	1.000	1.000					
30°	0.998	0.999	1.000				
45°	0.995	0.996	0.999	1.000			
60°	0.992	0.994	0.997	1.000	1.000		
75°	0.995	0.996	0.999	1.000	0.999	1.000	
90°	0.993	0.993	0.990	0.985	0.980	0.985	1.000

**Table 3:** The correlation between zenithal-average spectral radiances and different azimuth angles.

Azimuth	0°	15°	30°	45°	60°	75°	90°	105°	120°	135°	150°	165°
0°	1.000											
15°	1.000	1.000										
30°	1.000	1.000	1.000									
45°	1.000	1.000	1.000	1.000								
60°	1.000	1.000	1.000	1.000	1.000							
75°	0.999	0.999	1.000	1.000	1.000	1.000						
90°	0.999	0.999	0.999	0.999	1.000	1.000	1.000					
105°	0.997	0.997	0.998	0.998	0.999	0.999	1.000	1.000				
120°	0.996	0.996	0.996	0.997	0.998	0.998	0.999	1.000	1.000			
135°	0.994	0.994	0.994	0.995	0.996	0.997	0.998	0.999	1.000	1.000		
150°	0.992	0.992	0.993	0.993	0.995	0.996	0.997	0.999	0.999	1.000	1.000	
165°	0.991	0.991	0.991	0.992	0.993	0.995	0.996	0.998	0.999	1.000	1.000	1.000

**Table 4:** The correlation of spectral radiance at 12:00 and 14:00.

		12:00		
		Max	Middle	Min
14:00	Max	0.993	0.977	0.986
	Middle	0.966	0.997	0.957
	Min	0.993	0.972	0.991

**Table 5:** The correlation between the spectral radiances of lamps and skylight.

	Metal-Halide Lamp	Xenon Lamp	Tungsten Halogen Lamp	LED Lamp
Correlation Coefficient	0.57	0.81	0.10	-

**Table 6:** The parameters of the LightTools model.

Parameters	Value
Radius of the surface of lamps	25 mm
Beam angle	30°
Intensity	Normalized intensity distribution of each lamp
Exitance	Exitance distribution on the surface of lamp
Spectral characteristics	Normalized spectral exitance
Lumens	Lumens of each lamp
Zenith	30°/45°/60°/75°/90°
Azimuth	Every 45°/30°/30°/15°/15°
Radius of lamp array	1650 mm

**Table 7:** Components of skylight irradiance from different zeniths on 22 June.

Irradiance (μW/cm^2^)		Zenith (°)					Total Irradiance (μW/cm^2^)
		30	45	60	75	90	
Solar zenith (°)	18.56	3905	3293	2419	1434	191	11,244
	25.00	3778	3625	2722	1599	210	11,934
	52.62	1733	2786	3463	1975	257	10,214
	80.00	668	1055	1422	1487	222	4979

**Table 8:** The design of the lamp array with different working modes.

Layer No.	Number of Lamps	Zenith of Lamps (°)	Azimuth Interval (°)	Lamp Status (Irradiance (μW/cm^2^))		
				Mode 1 ^1^	Mode 2 ^2^	Mode 3 ^3^
				(11,047)	(9762)	(5031)
1	8	60	45	All on	1/2 on	1/4 on
2	12	45	30	All on	3/4 on	1/4 on
3	12	30	30	All on	All on	2/3 on
4	24	15	15	3/4 on	All on	2/3 on
5	24	0	15	All on	All on	All on
Relative deviation of irradiance				0.018	0.034	0.036

^1^ Mode 1: solar zenith 15°~40°; ^2^ Mode 2: solar zenith 40°~65°; ^3^ Mode 3: solar zenith 65°~80°.

**Table 9:** The irradiance of different modes.

Irradiance (μW/cm^2^)	Zenith (°)					Total Irradiance (μW/cm^2^)
	30	45	60	75	90	
Mode 1 MODTRAN	3905	3293	2419	1434	191	11,244
Mode 1 simulated	2997	3653	2465	1727	140	10,982
Mode 2 MODTRAN	1733	2786	3463	1975	257	10,214
Mode 2 simulated	1499	2740	2465	2302	140	9146
Mode 3 MODTRAN	668	1055	1422	1487	222	4854
Mode 3 simulated	749	913	1642	1535	140	4979
